# Research and development of Zika virus vaccines

**DOI:** 10.1038/npjvaccines.2016.7

**Published:** 2016-07-28

**Authors:** Brian E Dawes, Claire A Smalley, Bethany L Tiner, David WC Beasley, Gregg N Milligan, Lisa M Reece, Joachim Hombach, Alan DT Barrett

**Affiliations:** 1 Department of Microbiology and Immunology, University of Texas Medical Branch, Galveston, TX, USA; 2 Department of Pathology, University of Texas Medical Branch, Galveston, TX, USA; 3 Sealy Center for Vaccine Development, University of Texas Medical Branch, Galveston, TX, USA; 4 World Health Organization Collaborating Center for Vaccine Research, Evaluation and Training on Emerging Infectious Diseases, University of Texas Medical Branch, Galveston, TX, USA; 5 Institutional Office of Regulated Nonclinical Studies, University of Texas Medical Branch, Galveston, TX, USA; 6 Department of Pediatrics, University of Texas Medical Branch, Galveston, TX, USA; 7 Initiative for Vaccine Research, Department of Immunization, Vaccines and Biologicals, World Health Organization, Geneva, Switzerland

## Abstract

Zika virus (ZIKV) is a member of the family Flaviviridae, genus *Flavivirus,* and is transmitted by *Aedes sp.* mosquitoes. There are three genetic lineages of ZIKV: the East African, West African and Asian lineages. Until recently, Zika fever (ZF) has normally been considered a rare, mild febrile disease, but reports since 2012 have shown potentially severe complications associated with ZIKV infection, including microcephaly and Guillain–Barré syndrome. There are no licensed vaccines for ZIKV; however, many vaccine platforms/approaches that have been utilised for other flavivirus vaccines are being applied to ZIKV. Given the current outbreak of ZIKV in the Americas with its associated risks to pregnancy, we summarise what is known about the virus, how knowledge of currently licensed flavivirus vaccines can be applied to ZIKV vaccine development and the assessments of potential challenges for ZIKV vaccine testing and evaluation.

## Zika virus emergence, transmission and disease

Zika virus (ZIKV) was first isolated from a sentinel Rhesus macaque in the Zika forest of Uganda in 1947.^1–3^ The first isolations of ZIKV from humans occurred in Uganda and Nigeria in 1952.^4^ ZIKV is an enveloped, positive-sense, single-stranded RNA virus in the genus *Flavivirus,* family Flaviviridae, which includes other medically important mosquito-borne viruses such as dengue (DENV), Japanese encephalitis (JEV) and yellow fever (YFV).^2,3,5,6^ The ZIKV genome consists of 5′ and 3′ noncoding regions and a single open reading frame encoding a polyprotein that is co- and post-translationally processed to generate three structural proteins (capsid (C), precursor of membrane (prM) and envelope (E)), and seven non-structural (NS) proteins (NS1–NS5) in the gene order: 5′-C-prM-E-NS1-NS2A-NS2B-NS3-NS4A-NS4B-NS5-3′.^6^ Serological studies indicate that there is only one serotype of ZIKV, i.e., it is like JEV and YFV but different from DENV, which has four serotypes. ZIKV is most closely related, both phylogenetically and antigenically, to Spondweni virus (SPOV), which is also a human pathogen.^3,5,7^ Three genetic lineages of ZIKV have been identified that separate geographically: the East African, West African and the Asian lineages.^2,5–8^ Until the early 2000s, its geographic distribution was limited to the equatorial belt around Africa and Asia with circulation predominantly in wild primates and *Aedes sp.* mosquitoes, with rare ‘spillover’ infections in humans.^9^

## Recent ZIKV outbreaks

In April and May 2007, the first identified outbreak of Zika fever (ZF) occurred in the Federated States of Micronesia (Yap Island).^2,10,11^ From the 2000 census data, Yap had a population of 7,391 people. Duffy *et al.* identified 185 cases of suspected ZIKV disease. Of those, 49 (26%) were confirmed and 59 (32%) were probable cases. It was determined that about three quarters of Yap residents (~5,000 persons^12^) were infected with ZIKV, and more than 900 people had illness attributable to ZIKV infection.^13^ ZIKV epidemic activity was reported in Gabon during a concomitant chikungunya (CHIKV)/DENV outbreak in 2007 that involved ~20,000 cases.^12,14^ In 2013–2014, a large epidemic occurred in French Polynesia, which infected ~11% of the population.^2,15,16^ This epidemic spread throughout the Pacific to New Caledonia, Vanuatu, the Cook Islands and the Solomon Islands.^2,3,11,17^ The virus was then introduced to Easter Island in 2014.^18^ Zanluca *et al.*^19^ reported the first identification of ZIKV as the causative agent of an outbreak in northeastern Brazil in early 2015. That report represents the first autochthonous transmission of ZIKV in that country. By May 2015, ZIKV was reported by several other Central and South American countries in the Caribbean.^11^ From 1 January 2007 to 23 March 2016, ZIKV transmission was documented in 61 countries and territories (the outbreak is over in four of these countries). Autochthonous ZIKV transmission has been reported in 34 countries and territories of the Americas.^20^

## ZIKV Asian lineage strains associated with recent outbreaks

The 2007 outbreak in the Federated States of Micronesia was due to a ZIKV strain that was genetically related to the Asian lineage,^15,32^ and the strains isolated during the large epidemic in French Polynesia also belonged to the Asian lineage.^16^ However, based on limited numbers of genomic sequences available, these strains were more closely related to strains previously isolated in South East Asia than the strain isolated from the 2007 outbreak.^21,22^ ZIKV strains isolated from Brazil and the Americas, including those isolated from Easter Island, are closely related to the strains isolated during the outbreaks in French Polynesia, within the Asian lineage.^2,11,19,23^ The strains isolated in Brazil share more than 99.7% and 99.9% of nucleotide and amino-acid identity with the Asian strains, respectively.^24^ Furthermore, there is evidence that ZIKV is continuing to evolve in the Americas.^8^ There are a number of papers examining the evolution of ZIKV in the Americas and the apparent increased virulence of the virus. However, there are probably insufficient genomic sequences with information on passage history, origin and clinical history to make conclusions at this time. Faria *et al.*^11^ have suggested that ZIKV was introduced into the Americas in the second half of 2013 using a limited number of strains in their analysis, including 23 complete and partial genomic sequences. However, there are over 70 ZIKV genomic sequences available now and a subsequent paper has provided evidence that ZIKV was in Haiti in 2014.^25^ In addition, Zhu *et al.* reported some genomic changes in their comparison of pre-epidemic African and Asian strains (from mosquito and human isolates). They found a number of amino-acid substitutions throughout the genome and a conformational change in the SL1 structure at the 3′ noncoding region of the epidemic ZIKV strain. There was a possible recombination detected of a NS2B fragment between the Asian lineage of ZIKV and SPOV.^26^ Further investigations are required to elucidate how these changes may be influencing Zika’s overall virulence.

## ZIKV vectors and transmission

Many *Aedes* species have had a role in ZIKV transmission and are considered competent vectors including the African species *A. africanus*, *A. apicocoargenteus*, *A. luteocephalus*, *A. furcifer*, *A. vitattus* and *A. aegypti*.^3,6,27,28^ In Asia, a sylvatic cycle has not been identified but *A. aegypti*, *A. hensilli* and *A. albopictus* have been shown to be vectors.^3^ In areas with low populations of non-human primates, humans have been suggested as the primary amplification hosts,^13^ with sporadic human ZF cases being reported from the 1960s to 2007,^28^ involving only 14 clinical cases. Furthermore, there is serologic evidence of infection in forest-dwelling birds, horses, cattle, ducks, bats, elephants, goats, hippopotamuses, impala, kongoni, water buffalo, sheep, wildebeest, rodents and zebras.^5,21,29–31^ There are several other potential mechanisms of transmission, i.e., by blood transfusion, sexual transmission or perinatal transmission;^5,31–33^ however, these are believed to be relatively rare when compared with mosquito-borne transmission. Evidence to date indicates a low and transient viraemia in humans, and therefore it is unclear how the virus has managed to spread so widely and quickly in the Americas. Nonetheless, it should be remembered that, at present, there is limited information on the mosquito–vertebrate transmission cycle, particularly in the Americas.

## Clinical signs and symptoms of ZF compared with SPOV, DENV and CHIKV fevers

As stated above, SPOV is both antigenically and phylogenetically the most closely related virus to ZIKV, and it causes a febrile illness with reported symptoms of a brief fever, headache and malaise. Other reported symptoms include a fine maculopapular rash, conjunctivitis and photophobia.^37^ Similarly, ZIKV infection of humans has generally been considered an acute self-limiting febrile illness characterised by sudden onset of fever, maculopapular rash, arthralgia, retro-orbital pain and conjunctivitis. In some cases, symptoms include myalgia, headache and vomiting.^16,17,38,39^ Clinical presentations of ZF also resemble those that are caused by DENV and the alphavirus CHIKV^2,3,16,38^ and may last up to 10 days (acute phase).^13,28^ However, in contrast to dengue fever (DF), ZF is less severe than DF with headache and malaise being less intense, and signs of haemorrhage and/or shock (as seen for DENV infections) have not been reported.^13,16,17^ Furthermore, conjunctivitis is often present in ZF, but arthralgia is less pronounced.^13,16^ CHIKV presents with high fever, rigors, headache, photophobia, maculopapular rash and severe joint pain;^40^ ZIKV has not been reported to produce rigors or incapacitating joint pain.^41^ Another report described rash presenting 3–5 days after the febrile illness in ZF, but six patients had light asthenia and mild fever 2–3 days before the rash was observed.^17^ The incubation period is thought to be 3–14 days.^42^ Laboratory data include transient leukopaenia and, in some cases, thrombocytopaenia.^16,42^ There are estimations that 80% of ZIKV infections are asymptomatic,^39^ and severe ZIKV cases involving hospitalisation are uncommon and deaths are rare.^41^

## ZIKV and congenital abnormalities

While ZF has normally been considered to be a mild febrile disease, the full spectrum of pathology is not completely known but has recently expanded to include several neuropathies. Recent reports indicate potentially severe complications associated with ZIKV infection, including microcephaly, central nervous system malformations and Guillain–Barré syndrome (GBS).^17,38,43–45^ Zika congenital syndrome is the term used to refer to severe congenital malformations in humans infected by ZIKV. It describes features of congenital microcephaly: facial disproportionality and *cutis girata*, in addition to hypertonia or spasticity, hyper-reflexia, irritability, tremors and convulsions.^46^ In addition, severe microcephaly and other brain abnormalities observed in many infants were consistent with ZIKV infection occurring during the first- or early-second trimester of pregnancy. In the 2015 Brazilian outbreak, there were accounts of an increasing number of infants born with microcephaly in ZIKV-affected areas. To obtain data concerning pregnancies (e.g., exposure history, symptoms and laboratory tests), physical examinations of infants, and any additional studies, the Brazilian Society of Medical Genetics established the Zika Embryopathy Task Force. The task force reviews all incident cases of microcephaly as well as infants whose mothers are suspected of having ZIKV infection. The focus of the Task Force is to investigate the possible association of microcephaly with ZIKV infection during pregnancy and establish a standardised registry for incident microcephaly cases.^47^ As of 17 March 2016, a total of 6,671 microcephaly cases in Brazil are suspected to be associated with a ZIKV infection. Investigations have been concluded for 2,212 cases and 863 were confirmed for evidence of ZIKV infection. In addition, Ventura *et al.*^45^ have reported ophthalmic data from three children in Brazil with microcephaly born after the ZIKV outbreak. Here the infants had macular pigment mottling, foveal reflex loss, macular neuroretinal atrophy (one child) and cerebral calcifications (detected by CT scans) due to presumable intrauterine ZIKV infection.^45^ French Polynesian health authorities reported an increase in central nervous system malformation in fetuses and newborns during the ZIKV outbreaks. In that report, sera from 4 of these 17 pregnant women were positive by IgG for flavivirus, which may suggest asymptomatic or subclinical infections.^48^ ZIKV has been reported in Colombia since October 2015, and there have been over 30,000 cases of reported ZIKV infections where more than 5,000 of these cases were in pregnant women. As of 30 April 2016, there are no reports of associated cases of microcephaly in that country.^49^ In April 2016, because of the available evidence, the CDC concluded that there was sufficiently robust scientific evidence to establish a causal relationship between prenatal ZIKV infection and microcephaly and other serious brain anomalies.^50,51^

## ZIKV and GBS

GBS is an autoimmune disease characterised by progressive muscle weakness that can result in respiratory failure.^52^ Since October 2015, five countries or territories have reported increased GBS^53^ associated with ZIKV transmission in those areas. Current evidence suggests that the incidence of GBS is 8–10-fold higher in ZIKV IgM-positive individuals compared with the uninfected population. During the outbreak in French Polynesia, 73 cases of GBS and other neurologic conditions were reported among all ages of people.^38^ One study estimated that the rate of GBS after ZIKV infection was ~1 per 4,000 infections. Patients with Zika-associated GBS deteriorate rapidly with the average length of intensive care unit stay as 35 days.^52^ There have been unusual increases in reported GBS cases in the Americas, with Brazil reporting 121 cases, Venezuela describing 15 cases, El Salvador having 46 cases and Martinique reporting 2 confirmed cases.^38,54^ A new report from the recent American Academy of Neurology 2016 Annual Meeting describes a potential link between acute disseminated encephalomyelitis (ADEM) and ZIKV. Between December 2014 and June 2015, six patients in Pemambuco, Brazil, presented in the hospital with fever, rash, pruritus, myalgia, arthralgia and conjunctival hyperaemia. Two patients presented with ADEM and four with GBS. All six cases were positive for ZIKV. This is the first report of ADEM associated with the virus. Both ADEM and GBS are immunologic conditions triggered by infections. These reports of ADEM and GBS suggest that ZIKV may be triggering an immunological response causing these neurological conditions.^55^ Such immunological responses might reflect putative immunological cross-reactivity to glycolipids or peptide sequences shared between virus and host, or mechanisms such as epitope spreading, or bystander activation in genetically susceptible hosts. Alternatively, GBS may develop mainly as a consequence of the neurotropism of ZIKV.^56–58^ Nevertheless, links between ZIKV infection and GBS have not yet been proven since the ECDC Rapid Risk Assessment published their report on 10 December 2015.^38^ This is not surprising because GBS can be secondary to other immune events and other virus infections.^59^ At present, the relationship between ZIKV infection and GBS is incompletely understood, and more studies are needed to firmly establish a mechanism-based link.

## ZIKV diagnostic procedures

The presumptive diagnosis of ZIKV is typically clinical with confirmatory laboratory tests performed using serum, saliva and/or urine samples. If samples are collected within 1–3 days of fever onset, NS1 antigen may be detected in serum and reverse transcription PCR (RT-PCR) can be used to detect a specific region of the viral genome that includes NS5.^43^ ZIKV RNA can also be detected in both urine and saliva samples collected within the first 3–5 days of fever onset.^22–24^ Current testing recommendations are to obtain RT-PCR results from urine or saliva within the first 5 or 6 days of illness.^5^ Serological tests, such as enzyme-linked immunosorbent assays and immunofluorescence assays can be performed to detect anti-ZIKV IgM and IgG antibodies.^62,63^ For confirmation of enzyme-linked immunosorbent assay-positive and RT-PCR-negative samples, a plaque reduction neutralisation test^5^ can be performed at reference laboratories. Nevertheless, secondary flavivirus infections potentially complicate plaque reduction neutralisation test-based diagnostics because of the induction of broadly cross-reactive antiflavivirus antibody responses^13^ (e.g., including ZIKV there at least 10 flaviviruses in Brazil). The World Health Organization (WHO) has established an Emergency Use and Assessment Listing for diagnostic tests in response to which 10 tests, both PCR- and serology-based, have been submitted (http://www.who.int/diagnostics_laboratory/eual-zika-virus/160520_weekly_update.pdf?ua=1).

## Current ZIKV infection management

There are neither antiviral treatments nor vaccines for ZIKV available currently.^9,64^ In addition, there are no standardised reference reagents (antigens or antibodies), although these are in development. Treatment is thus symptomatic such as treating pain and fever with the use of acetaminophen (paracetamol).^64^ Current preventions include personal protection, e.g., mosquito repellent, avoidance of mosquito bites (especially by pregnant women and ZIKV-infected individuals), wearing long pants and/or shirts with long sleeves, screens, bed nets and mosquito surveillance and control methods.^5,32,64^

## Current vaccine development efforts

Although there are no licensed vaccines for ZIKV,^9^ many vaccine platforms/approaches that have been utilised for vaccine research for other flaviviruses are being applied to ZIKV. Table 1 shows platforms/technologies being used in nonclinical development of flavivirus vaccines,^65^ and many groups are investigating the same technologies for a ZIKV vaccine.

## Protective immunity against ZIKV

The current understanding of protective immune responses to ZIKV is limited, and is derived primarily from human data following infection and from comparison with other flaviviruses. Epidemiological evidence suggests that there is a period of time following acute infection in which patients are immune to reinfection, although the long-term durability of this protection is not known.^13,15^ On the basis of evidence from other flavivirus infections, natural infection with ZIKV is likely to result in life-long immunity. Further evidence shows that for secondary (i.e., Zika following some other flavivirus) infections, at least a fourfold rise in neutralising antibody titre against ZIKV occurs between the acute to convalescent phase.^66^ In analysing patient serum from the 2013–2014 ZIKV outbreak in Micronesia, the predominant acute-phase antibody was IgM that was cross-reactive with a variety of flaviviruses, which transitioned to cross-reactive IgM and IgG antibodies in the convalescent phase.^62^ In April 2007, an epidemic ZIKV was noted on Yap Island. Some patients had detectable IgM levels as early as day 3 post onset of symptoms, and some produced neutralising antibody by day 5 after onset of symptoms as measured by neutralisation assays.^62^ Altogether, these data suggest that neutralising antibodies are essential for protection. This would be expected, as available data suggest that for three of the currently licensed flavivirus vaccines (YFV, JEV and tick-borne encephalitis (TBEV)), neutralising antibodies represent a correlate of protection and that a neutralisation titre of 1 in 5 or 1 in 10 is protective.^67^ For dengue, and the recently licensed recombinant live tetravalent vaccine, the situation is more complex and a correlate of protection has not been identified yet. Cytokine profiles taken from acute and convalescent human patient serum are indicative of the development of cell-mediated immunity reflected by increased levels of polyfunctional T helper-associated cytokines and chemokines.^16^ More work is required to define a relationship between cell-mediated immunity and immune protection against ZIKV, including for long-term immunity.

## Live attenuated flavivirus vaccines

Live attenuated vaccines (LAVs) offer protective immunity after one or a few doses because of multiplication of the vaccine in the host, which stimulates T and B cells.^68^ Although LAVs are effective, an important potential limitation is safety, as there is always a risk of reversion to virulence.^69^ However, LAVs against flaviviruses, such as JE SA14-14-2 or YF 17D, have had significant success, inducing robust protective immunity after one dose because of the capability of replication within the host, thereby imitating natural infection.^65^ Recently, chimeric recombinant LAVs for JEV and DENV have been licensed based on the YF 17D vaccine virus backbone with the YF 17D prM/E genes replaced by those of JEV and DENV, respectively.^70–72^ There is little doubt that a LAV for ZIKV would be a very effective vaccine; however, our understanding of ZIKV clinical disease is limited, and animal models are still in the discovery phase. Consequently, identifying an attenuated phenotype for a ZIKV LAV will be very demanding and the evaluation process likely complicated.

## Inactivated flavivirus vaccines

Inactivated flavivirus vaccines have been licensed for JEV, TBEV^8^ and Kyasanur Forest disease. Inactivated vaccines require multiple doses and very high virus titres/quantity of protein to induce a protective immune response. There is very little information available on the Kyasanur Forest disease vaccine.^73,74^ However, those licensed for JEV and TBEV^8^ require two doses and very high virus titres (10^8 ^p.f.u. equivalent per dose or ~6 μg purified, inactivated JEV proteins and 3 μg inactivated TBEV proteins) and are mostly adjuvanted with alum (250 mg aluminium hydroxide) to induce a protective immune response for 3–5 years. Clearly, development of inactivated ZIKV vaccines could follow the development path utilised for inactivated JEV and TBEV vaccines with a two-dose regimen to give protective immunity, and duration of immunity followed post licensure. In addition, adjuvants, including oil-in-water emulsions, have the potential for dose-sparing, and preliminary clinical data have been collected for inactivated flavivirus vaccines, including candidate dengue vaccines.

## Non-replicating, subunit flavivirus vaccines

There are no licensed flavivirus subunit, non-replicating vaccines. However, there are a number of candidates, including recombinant subunit vaccines and DNA vaccines, that are all promising technologies and may be suitable for application to ZIKV, including special populations.^65^ Nevertheless, as mentioned earlier, the non-replicating nature of these types of vaccines requires multiple doses and use of an adjuvant to establish and maintain immunity, which adds complexity to the roll-out of such vaccines.

Recombinant subunit vaccines produce robust immunity to specific antigenic epitopes; however, more work is required to determine which components of ZIKV stimulate neutralising antibody. On the basis of research with other medically important flaviviruses, the envelope (E) protein would be a critical component. Such vaccine development would be aided by the availability of structural data. Indeed, recent studies have reported the cryo-electron microscopic structure of ZIKV mature virions based on virus grown in Vero cells,^75,76^ demonstrating that the overall structure of ZIKV virions is similar to that of DENV. Furthermore, the structures of the E protein complexed with a neutralising monoclonal antibody Fab fragment^77^ and NS1^78^ (protein that induces complement-fixing antibodies) have been solved.

DNA vaccines offer great potential interest for relative ease of development and manufacture,^79^ and have provided a platform that induces robust cellular and humoral immune responses in small animals and non-human primates.^52^ Unfortunately, based on DENV they are yet to show promising results in limited clinical studies.^80^

## Animal models for ZIKV studies

There is no established animal model for ZIKV studies, which makes preclinical evaluation of candidate ZIKV vaccines in animals difficult. ZIKV infection results in a neurotropic and fatal disease in suckling and weanling immunocompetent mice following intracerebral inoculation.^1^ Histopathology of mouse brains shows both cellular degeneration and the development of inclusion bodies in the central nervous system.^27^ Immunocompetent animals, including rhesus monkeys, rabbits and guinea pigs, exhibit no clinical disease when infected; however, rhesus monkeys will develop transient viraemia and pyrexia.^1^ Immunocompetent animals develop viraemia and produce antibodies to ZIKV. As it has been possible to develop non-neurotropic disease for YF^81^ and DEN viruses^53, 54, 55, 82^ in immunocompromised mice, a number of groups have developed mouse models for ZIKV. Recently, five papers have described lethal ZIKV mouse models utilising mice deficient in either interferon (IFN)-α/β receptors (A129)^39,85,86^ or both IFN-α/β and IFN-γ receptors (AG129).^87,88^ The A129 mouse model appears to have limitations for vaccine studies as clinical signs are only seen in young mice, whereas ZIKV causes clinical disease in older AG129 mice, which would be more suitable for vaccine immunisation and challenge studies. However, more studies are required before conclusions can be made on potential mouse models. Nonetheless, based on studies with other flaviviruses, there are benefits and disadvantages of utilising immunocompromised mice for vaccine development.^83^ Whereas live vaccine candidates appear to induce a strong adaptive immune response, limited studies show that AG129 mice are not ideal to evaluate recombinant protein-based DENV vaccine candidates;^83,84^ however, further work is needed in this area. There are also studies underway to develop non-human primate models of ZIKV infection and *in utero* transmission.^89^

## Preferred characteristics of ZIKV vaccines for use in endemic areas and/or outbreak response

Predicting the future use of a licensed ZIKV vaccine is difficult in the absence of a better understanding of ZIKV epidemiology and transmission cycles. It is conceivable that ZIKV vaccine(s) could be a part of the routine vaccination schedule in endemic areas. An emergency use target product profile is being developed by WHO to respond to current or future major epidemics.

There are two possibilities of transmission cycles in the Americas, either the cycle is primarily human–mosquito–human (e.g., DENV) or it is zoonotic involving an animal–mosquito cycle with humans being incidental/dead-end hosts (e.g., JEV), and the approach to vaccination would likely differ depending on the transmission cycle. In the emergency context, candidate vaccines would likely be prioritised for women of childbearing age and pregnant women, given the association of ZIKV with microcephaly in neonates. Nonetheless, such vaccine development is very challenging.

Ideally, the vaccine(s) produced should be suitable for use and distribution in low- and middle-income countries, such as Brazil. This requires consideration by developers of issues such as thermostability, need for temperature control in the supply chain and cost of post-licensure clinical studies to evaluate the safety and efficacy of the final vaccine(s) in endemic countries that may lack infrastructure to support those studies. The definition of product characteristics in context of the target product profile for a ZIKV vaccine will aid in focusing development efforts and investment in response to the current outbreak.

## Challenges for development and testing of ZIKV vaccine candidates

There are more than 60 research institutes and companies working on products to combat the spread of ZIKV.^90^ The pathway to a ZIKV vaccine is still in the nonclinical stage.^91^ To date, there is only one published paper^92^ on ZIKV vaccine candidates, and very little information is available regarding induction of immunity against ZIKV in humans or animals. However, the related flavivirus, DENV, is the topic of intensive vaccine research with a recently licensed product and several vaccine candidates in clinical and preclinical development.^65,90^ Several of these vaccine platforms could potentially be applied to ZIKV vaccine development. Notably, ChimeriVax yellow fever-Japanese encephalitis vaccine has been licensed since 2010 (under the trade name IMOJEV) and the ChimeriVax-yellow fever dengue LAV vaccine from Sanofi Pasteur (trade name DENGVAXIA) containing DENV structural genes in a YFV backbone has completed phase III clinical trials and been licensed in several countries.^71,93^ Additional recombinant or chimeric LAV DENV vaccines have entered phase II or III trials, and purified inactivated, recombinant subunit and DNA vaccines have entered phase I trials.^93^ Suitability of these approaches for the target population(s) needs to be considered. Using one of these platforms utilising ZIKV antigens should be technically feasible and would potentially result in faster vaccine development because of the use of regulatory pathways established for these platforms during JEV and DENV vaccine development. Before these platforms enter clinical evaluation, nonclinical evaluation must be conducted, which is hampered by the lack of a validated animal model.

An additional gap in knowledge is the mechanism of protective immunity in ZIKV. Long-lasting protective immunity to natural ZIKV infection has not yet been demonstrated, but likely exists similar to that seen for other flaviviruses. Therefore, neutralising antibodies targeted to the E protein will likely have a major role in protective immunity, although cell-mediated responses will likely also be necessary. In addition, no standardised assays currently exist to measure ZIKV-neutralising antibodies. Ideally, criteria for standardisation of the key parameters for enzyme-linked immunosorbent assay and neutralisation assays should be established before clinical testing.

Once nonclinical studies have addressed the issues above and led to the production of a vaccine candidate, the implementation of phase II/III clinical trials may be logistically difficult because of the sporadic nature of arboviral outbreaks. In the past, ZIKV outbreaks have not typically remained sustained, and this may limit the ability to assess vaccine efficacy in large-scale field trials. This has been a problem for West Nile virus vaccine development. Depending upon the ongoing burden of endemic disease or occurrence of new large outbreaks where candidate vaccines can be evaluated, the definition of appropriate and measureable clinical end point(s) will also affect the design and feasibility of studies to demonstrate clinical efficacy. On the other hand, given the experience with other monovalent flavivirus vaccines, a licensure based on immunological data could be considered (e.g., induction of a particular titre of neutralising antibodies). Identifying a seroprotective neutralising antibody titre may be difficult. The potential role of antiflavivirus antibodies from previous flavivirus infection or vaccination will need to be addressed as well, as this has been noted to modify vaccine response,^91^ and may complicate assaying of ZIKV type-specific neutralisation titres in vaccines.

Another concern will be the use of ZIKV vaccine to protect pregnant women because of the association of ZIKV infection with fetal neurological defects such as microcephaly. Pregnant women are a special population, and therefore any ZIKV vaccine will need to undergo appropriate nonclinical and clinical safety testing before undergoing trials in pregnant women. An important consideration is the method of immunisation administration to this target population. Later phase clinical trials may investigate maternal immunisation; therefore, protection could be conferred to the pregnant women and their unborn fetuses. Administration of inactivated vaccines during pregnancy would provide passive protection to the baby via transfer of vaccine-induced immunoglobulin across the placenta.^68^ In the short-term and emergency context, the vaccination of pregnant women might be acceptable in the absence of a specific indication and based on a more restricted set of data, requiring a thorough risk benefit assessment, and the absence of a contraindication on the product label. In the longer term, one approach that may be considered is administration of a vaccine that gives long-term protection before childbearing age such that it would protect against congenital disease. Specifically, a LAV viraemia could stimulate protective immunity that is sufficiently robust to prevent any viraemia during subsequent infection, assuming that viraemia is a risk for infection of the fetus even in the absence of clinical disease in the mother.

For all vaccine candidates, the assessment of the risk of triggering GBS will constitute an adverse event of special interest, and will need to be studied throughout the development programme, and monitoring post registration.

Like West Nile virus and CHIKV before it, the emergence of ZIKV since 2007 and the explosive outbreak currently occurring in the Americas highlight the potential for relatively obscure arboviruses to rapidly and unexpectedly expand both their geographic range and their importance as agents of human disease. Whether this emergence and the occurrence of apparently severe diseases are because of virus, host or other factors remains to be determined. The global response to ZIKV will presumably leverage lessons learned from the response to the 2014–2015 Ebola virus outbreak in West Africa to facilitate rapid development and testing of new vaccine candidates and other products for prevention/treatment of infection or severe disease. Although many questions remain to be answered regarding the specifics of ZIKV infection and disease, the development and testing of ZIKV vaccines will benefit from recent experience with other flavivirus vaccine candidates that have utilised a wide range of platforms and provided insights into the induction of protective, long-lasting immunity to flaviviruses.

## Figures and Tables

**Table 1:** Examples of potential ZIKV vaccine strategies based on studies with other flaviviruses

*Technological approach*	*Antigen*^a^
Recombinant subunit vaccines	EDIII-p64k fusion proteins and EDIII-capsid fusion proteins expressed in *Escherichia coli*
	Bivalent 80E-STF2 fusion proteins expressed in baculovirus/insect cells
	E protein
	80 E protein
	EDIII protein expressed in *E. coli*
	
DNA vaccines	prM/E expressed from plasmid vector
	
VLP vaccines	prM/E
	EDIII-HBsAg VLPs or ectoE-based VLPs expressed in *Pichia pastoris*
	MVA-VLP
	
Recombinant chimeric live vaccines	YF 17D backbone
	DENV-2 backbone
	JE SA14-14-2 backbone
	Host range mutations
	Targeted mutation (2′-*O*-Methyltransferase mutant)
	DENV-4 backbone
	EDIII expressed from live-attenuated measles virus vector
	
Single round replicating viruses	E85 expressed from single-cycle VEE virus vector
	RepliVax
	
Virus-vectored vaccines	Live adenovirus 4/7 oral vector
Purified inactivated virus vaccines	Purified inactivated
	Purified inactivated virus (+Venezuelan equine encephalitis −particle adjuvant)

Abbreviations: DENV, dengue virus; MVA-VLP, modified vaccinia ankara-virus like particle; VEE, Venezuelan equine encephalitis; ZIKV, Zika virus.

a 80E and E85 refer to the N-terminal 80% and 85% of the E protein, respectively, which is the ectodomain of the E protein (also termed ectoE by some). EDIII is domain III of the ectodomain. prM/E is premembrane and envelope protein genes.
